# Rapid prediction of secondary neurologic decline after traumatic brain injury: a data analytic approach

**DOI:** 10.1038/s41598-022-26318-4

**Published:** 2023-01-09

**Authors:** Jamie Podell, Shiming Yang, Serenity Miller, Ryan Felix, Hemantkumar Tripathi, Gunjan Parikh, Catriona Miller, Hegang Chen, Yi-Mei Kuo, Chien Yu Lin, Peter Hu, Neeraj Badjatia

**Affiliations:** 1 Program in Trauma, Shock Trauma Neurocritical Care, University of Maryland School of Medicine, 22 S. Greene Street, G7K19, Baltimore, MD 21201 USA; 2 Department of Neurology, University of Maryland School of Medicine, Baltimore, USA; 3 Department of Anesthesiology, University of Maryland School of Medicine, Baltimore, USA; 4 Department of Epidemiology and Public Health, University of Maryland School of Medicine, Baltimore, USA

**Keywords:** Trauma, Predictive markers, Computational models, Brain injuries

## Abstract

Secondary neurologic decline (ND) after traumatic brain injury (TBI) is independently associated with outcome, but robust predictors of ND are lacking. In this retrospective analysis of consecutive isolated TBI admissions to the R. Adams Cowley Shock Trauma Center between November 2015 and June 2018, we aimed to develop a triage decision support tool to quantify risk for early ND. Three machine learning models based on clinical, physiologic, or combined characteristics from the first hour of hospital resuscitation were created. Among 905 TBI cases, 165 (18%) experienced one or more ND events (130 clinical, 51 neurosurgical, and 54 radiographic) within 48 h of presentation. In the prediction of ND, the clinical plus physiologic data model performed similarly to the physiologic only model, with concordance indices of 0.85 (0.824–0.877) and 0.84 (0.812–0.868), respectively. Both outperformed the clinical only model, which had a concordance index of 0.72 (0.688–0.759). This preliminary work suggests that a data-driven approach utilizing physiologic and basic clinical data from the first hour of resuscitation after TBI has the potential to serve as a decision support tool for clinicians seeking to identify patients at high or low risk for ND.

## Introduction

Traumatic brain injury (TBI) accounts for over two and a half million emergency department visits in the United States annually^1^ and is a leading cause for evacuation from austere military and civilian environments^2,3^. While many individuals recover fully, TBI results in enormous costs to society as the second leading cause of disability in the United States^1^. Acute trauma care has long stressed the importance of rapid assessment and skilled treatment during the “golden hour” of resuscitation, when interventions are most likely to prevent long-term morbidity and mortality^4^. Advances in point-of-care tools have aided in the rapidity by which systemic trauma patients can be prioritized, as with the Extended Focused Assessment with Sonography in Trauma (E-FAST) exam^5^; however, without analogous brain injury risk stratification tools, early triage and management of TBI patients remains challenging, especially in resource-limited austere environments^3^.

The primary triage concern, regardless of initial severity, is determining the likelihood of secondary neurological decline (ND), defined by clinical or radiographic worsening or need for neurosurgical intervention. An estimated 20% of moderate to severe^6^ and 5–10% of mild^7^ TBI patients experience ND, which is independently associated with worse outcome^8,9^. Appropriately timed medical and surgical interventions may prevent or mitigate secondary brain injury associated with ND, whereas prolonged monitoring and routine follow-up imaging may consume unnecessary resources in low-risk patients. Therefore, identifying individuals at highest and lowest risk of ND is a primary goal in initial TBI triage and resuscitation.

However, during the first hour of TBI resuscitation, risk stratification is based off of rudimentary data including the neurologic exam, static vital signs (VS), and clinical judgment^9^. While none have translated to standard clinical practice, there is growing interest in point-of-care technologies including ultrasound, pupillometry, and blood biomarkers for early TBI triage^3,10,11^. Tools utilizing dynamic VS for prediction of ND are particularly attractive given ubiquitous VS monitoring across settings. Further, non-specific early warning scores using static VS to predict clinical deterioration already have shown some utility in TBI patients^12^. More nuanced physiologic markers and trends—detectable by machine learning (ML) but not routine clinical observation—may better characterize and predict patient trajectories during the golden hour of TBI resuscitation by providing insight into autonomic nervous system dysfunction associated with impending ND. ML is emerging as a useful tool to predict deterioration and need for intervention in numerous clinical scenarios^13,14^, including trauma resuscitation^15,16^. While IMPACT and CRASH models can aid in TBI prognostication by using early admission data to predict mortality and 6 month outcome^17,18^, there remains a critical need to predict more proximate potentially actionable events that might contribute to longer term outcomes. This is the goal of our work aiming to predict early ND, to support early triage and clinical decision making.

We previously demonstrated that analysis of photoplethysmography (PPG) and electrocardiography (ECG) waveform and variability data during the first 15 min of resuscitation could better predict ND than clinical variables alone in a predominantly mild TBI cohort^19^. In the present study, we expand upon these findings in a larger TBI cohort with the goal of identifying physiological markers within the first hour of resuscitation, from data accessible to pre-clinical or austere environments, to aid in the clinical determination of ND risk over the subsequent 48 h.

## Materials and methods

### Patient selection, study design, and data acquisition

This is a retrospective cohort study of consecutive TBI patients cared for in the Trauma Resuscitation Unit at the R Adams Cowley Shock Trauma Center at the University of Maryland Medical Center. Included patients met the following criteria: 1. Diagnosis of survivable TBI [Head Abbreviated Injury Score(AIS) 1–5]^20^; 2. Direct admission from scene of injury (while a reliable exact time from incident to admission was not available for these patients, the majority arrive within one hour of injury); 3. Age > 18 years old. 4. No major systemic injuries (Thoracic or Abdominal AIS $\le$ 1); 5. No confounding active substance abuse (toxicology screen negative for cocaine and opiates). Patients were then excluded for the following: 1. Hospital length of stay less than 48 h; 2. Missing or insufficient physiologic data, defined as < 20 min of the first hour recorded. Baseline demographic and clinical data was obtained from the institutional trauma registry.

Continuous physiologic data was obtained from ECG, PPG, and arterial blood pressure (ABP) waveforms collected via BedMaster® (Excel Medical Electronics Inc., Jupiter, FL) VS collection system for 60 min beginning at the time of hospital arrival^21^. Trended VS such as heart rate (HR), peripheral capillary oxygen saturation (SpO2), and respiratory rate (RR) were collected every two seconds (0.5 Hz), and ECG, PPG, and ABP waveforms were collected at 240 Hz. All head computed tomography (CT) scans performed within the first 48 h were independently rated using Rotterdam and Marshall scoring systems^22,23^ by study investigators (NB, GP, JP), who were blinded to demographic and outcome data.

This study was performed as part of the Real-Time Vital Sign Assessment to Predict Neurological Decline After Traumatic Brain Injury (RAPID-TBI) study, funded by the Department of Defense (FA8650-18-2-6H18). The study was approved by the institutional review board of the University of Maryland, Baltimore (HP-00060944), which waived the need for informed consent. All research was performed in accordance with the Declaration of Helsinki^24^ and the Health Insurance Portability and Accountability Act^25^.

### Outcome measures

The primary outcome was ND between one and 48 h of admission. As in previous work^19^, ND was a binary variable defined by the occurrence of one or more clinical or radiographic worsening events. Clinical ND was defined by a spontaneous decrease in Glasgow Coma Scale (GCS) by 2 or more points, loss of pupillary reactivity, development of pupillary asymmetry of $\ge$ 2 mm, treatment of raised intracranial pressure, or need for neurosurgical intervention. Hourly clinical exam findings were obtained from review of electronic medical record (EMR) (EPIC Systems Cooperation) nursing flowsheets. Each case of clinical ND was reviewed by study investigators to ensure clinical decline was not attributable to use of sedatives, analgesics, neuromuscular blockade, or dilated ophthalmologic exams. Clinical ND events were subclassified as neurosurgical ND when progressive cerebral edema resulted in a neurosurgical intervention, the timing of which was ascertained via EMR review of operative notes. These clinical ND events were adjudicated via consensus among reviewing investigators (NB, GP, JP).

Radiographic ND was defined by a ≥ 1 point worsening of the Rotterdam^23^ severity score from initial to follow-up CT. While often insensitive to findings such as hemorrhage expansion, the Rotterdam is reliably associated with outcome after TBI^23,26^, and was therefor selected over other more granular radiographic features to ensure the clinical relevance of radiographic ND. As part of routine institutional practice all patients with moderate to severe TBI underwent follow up head CT imaging at 6 and 24 h after admission. Patients presenting with mild TBI (GCS 13–15) underwent CT imaging based on findings of initial scan and/or presenting mechanism of injury. Interrater reliability of Rotterdam scores assessed by three authors was calculated using data from 180 patients (approximately 20% of 905 total patients). The Cronbach alpha (95% confidence interval) was 0.966 (0.949, 0.979), and there was 100% interrater agreement regarding cases of radiographic ND (where Rotterdam worsened by ≥ 1 point).

If a patient experienced more than one ND event within the first 48 h, then time of ND was defined by the earliest event. Individuals who experienced ND within the first hour of admission were excluded from analysis given desire to use the first hour of data to forecast future ND. Additional secondary outcomes were collected via review of trauma registry data. These included hospital length of stay, mortality, and discharge destination.

Descriptive and univariate statistics were performed comparing presenting demographic, clinical, and injury characteristics and hospital outcomes across patients with and without ND. The Wilcoxon rank sum test was used for ordinal variables and the Chi-squared test for categorical variables using a threshold of *p* < 0.05 for statistical significance.

### Physiologic data processing

High-fidelity PPG and ECG waveform and trended VS data collected during the first hour of admission was pre-processed by removal of extreme values deemed outside of normal physiologic ranges (HR > 200 bpm, SBP > 250 mmHg, DBP > 200 mmHg) and extreme outliers (based on a moving median with a window of 30 s) before extraction of features^27^. The ECG and PPG signals were smoothed with a robust discretized spline smooth filter to improve the signal-to-noise ratio^28^. These filtering steps may be automatically applied in a real time prospective manner. As previously described^15^, VS features including mean, standard deviation (SD), median, interquartile range (IQR), dose above and below thresholds, and first, second, and third quartiles of HR, systolic blood pressure (SBP), diastolic blood pressure (DBP), shock index (SI = HR/SBP), and SpO2 were extracted from trended VS data. PPG and ECG-based heart rate variability (HRV) time and frequency domain features were calculated using standard definitions based on the Task Force of the European Society of Pacing and Electrophysiology^29^ (Supplemental Table 1). Frequency domain features were calculated using three distinct popular methods, each with their own strengths and weaknesses^30^, including Welch’s method of averaging periodograms from overlapping intervals^31^, analysis of least-squares based Lomb periodograms^32^, and parametric autoregressive modeling^33^. Non-linear dynamics HRV features included measures of entropy, Poincare plots, and fractal analyses^34^.

Our prior work suggested that clinical measurements, such as admission GCS, could improve prediction of ND^19^. However, adding these variables requires manual evaluation with clinical expertise. To build a fully automated physiologic model, we created GCS and Injury Severity Score (ISS)^20^ estimation models that use only VS variables, derived from a set of non-overlapping patient data collected prior to this study^35,36^. These models were used to generate additional variable inputs for ND prediction models, namely, model outputs for the prediction of specific GCS and ISS scores^36^. The GCS estimation model generates the probability that a patient’s GCS at admission is a particular value from 3 to 15. The ISS estimation model outputs the probability that a patient’s ISS falls into one of five categories: 1 (1–4), 2 (5–8), 3 (9–15), 4 (16–24), and 5 (25–75). This is a form of transfer learning^37^, where lessons learned from large-scale physiologic data predictions are transferred as a substitution for the value being predicted into a new model.

### Prediction model development

Three ND prediction models were created based on different kinds of predictor variables (clinical, physiologic, and combined models). The clinical model utilized presenting demographic information (age, sex, race/ethnicity), injury characteristics (type, mechanism), and clinical exam (arrival GCS as documented by TRU staff), all of which would have been known within the first hour of hospital presentation. The physiologic model utilized VS features from the first hour of admission, including the transfer learning variables described above. The combined model included both clinical and physiologic features from the first hour of admission.

To study the time-to-event data and handle model nonlinearity and missing values, the gradient boosted survival tree (GBS) model was used^38^. The GBT uses an ensemble of decision trees to sequentially learn the error left over by the previous tree. It has been demonstrated to outperform other machine learning methods in CPH analysis, such as random forest or support vector machine^39^.

To simplify model training and improve interpretation (e.g. variable importance), we used the Maximum Relevance Minimum Redundancy (MRMR) method to select features^40^. This method attempts to find a subset of features that maximize the association while minimizing the redundancy (measured by mutual information) between the features and the outcome. We selected 20 features that satisfied the MRMR.

During training, to prevent over-fitting, we tuned model hyperparameters including total number of trees, maximum tree depth, learning rate, and percentage of sampled variables. Through fivefold cross-validation, optimal hyperparameters were identified via random grid search. SHapley Additive exPlanations (SHAP) values were calculated and displayed graphically in order to describe each variable’s contribution to ND prediction^41^ and improve model interpretability^39^.

Model performance was described using time-dependent receiver operating characteristic (ROC) areas under the curve (AUC) and their 95% confidence intervals (CI). Overall (time-independent) performance for each model was described by its concordance index (C-index) and 95% CI^42,43^. Model development and statistical analyses were performed by Extreme Gradient Boosting (XGBoost), an efficient Python implementation of GBT (xgboost, v1.3.3)^44^. Missing waveform data was missing completely at random^45^ and occurred in 20 patients (approximately 2%). It was handled by imputing with the most frequent values.

As an exploratory analysis, we assessed whether our ND prediction model could also predict in-hospital mortality. To this end, we used the ROC analysis to evaluate the association between ND model output and in-hospital mortality. This model’s performance was described by the AUROC and its 95% CI.

## Results

### Baseline characteristics

**Figure 1:**
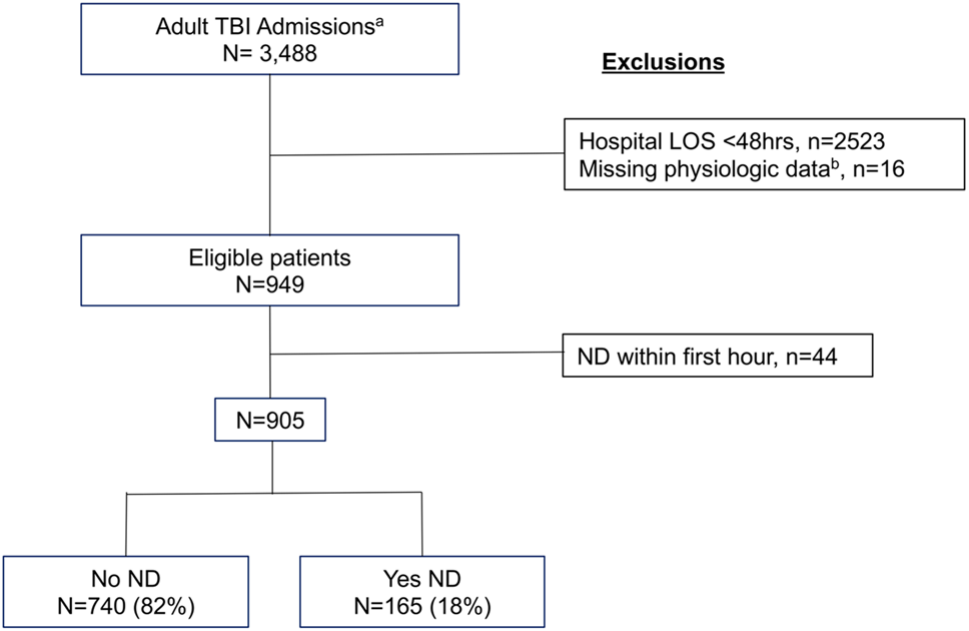
^a^All cases identified from trauma registry with Head AIS 1–5, Thoracic/Abdominal AIS $\le 1$, and toxicology screen negative for opiates and cocaine, from Nov 2015–Jun 2018. ^b^Cases included if $\ge$ 30% of first hour continuous physiologic data was available for analysis. TBI = traumatic brain injury; LOS = hospital length of stay; ND = neurologic decline.

**Figure 2:**
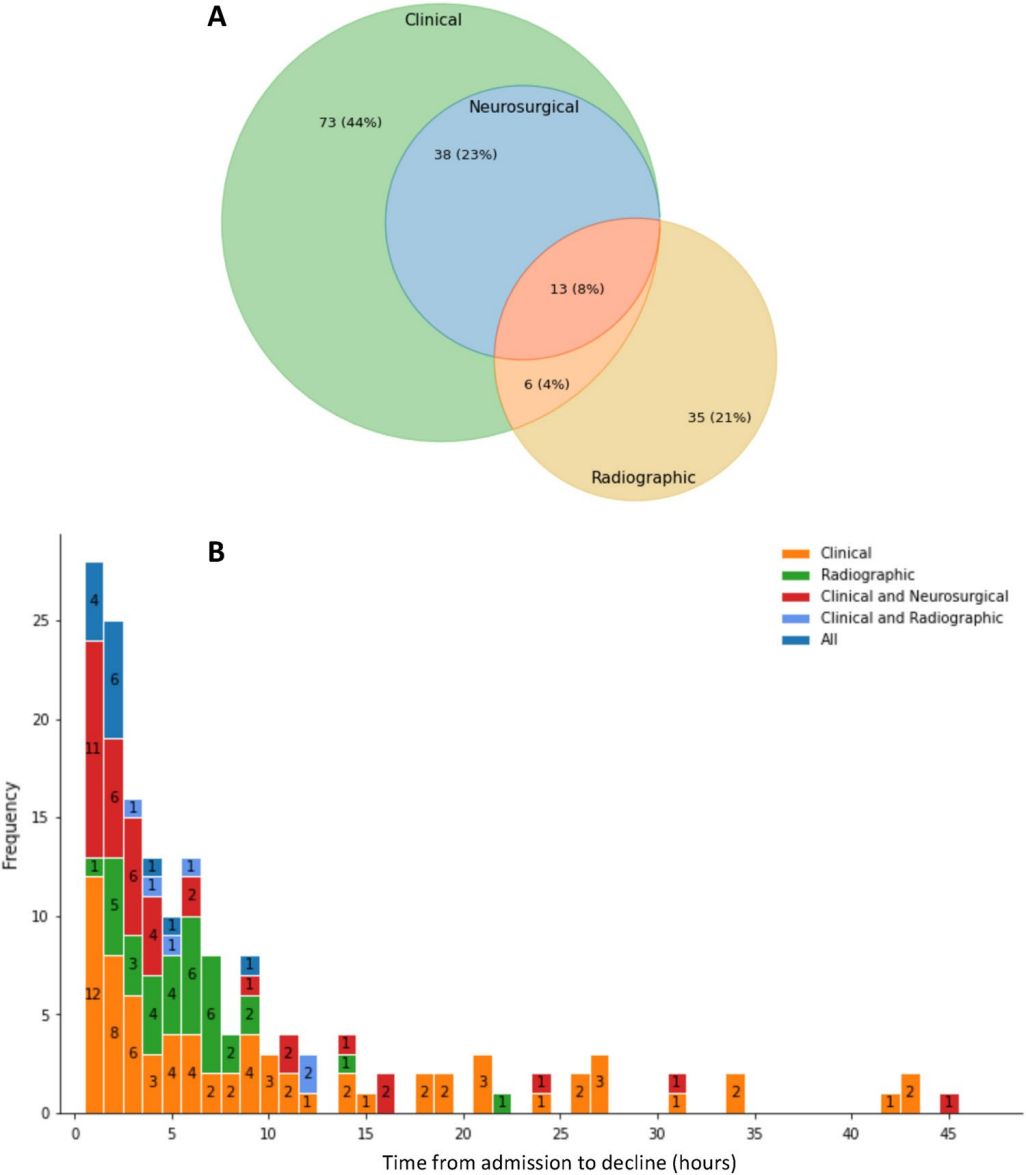
A. Ven diagram displaying the prevalence of ND events by subtype. B. Temporal distribution of ND, with y-axis representing the number of patients experiencing ND during each hour, as denoted by the x-axis, color-coded by ND subtype.

Admission characteristics of patients grouped by primary outcome are described in Table 1. Of 905 eligible TBI cases, 165 (18%) experienced ND of any kind within 48 h of admission (Fig. 1). The type and timing of ND events are displayed in Fig. 2; clinical ND occurred in 130 (79% with ND; 14% overall), radiographic ND in 54 (33% with ND; 6% overall), and neurosurgical ND in 51 patients (31% with ND; 6% overall). More than one ND type occurred in 57 patients (35% with ND; 6% overall), with all three types occurring in 13 (8% with ND; 1% overall). The median time to ND was 5 h (IQR 3–10). Several arrival VS demonstrated small but statistically significant associations with ND, including temperature and oxygen saturation. Clinical factors found to be associated with ND on univariate analysis included penetrating injury (*P* < 0.001) and lower GCS (*P* < 0.001).

**Table 1:** Demographic, clinical, and injury characteristics.

	Neurologic decline		*p*-value
	Yes	No	
	N = 165 (18)	N = 740 (82)	
**Age**^1^	57 (38, 75)	54 (33, 71)	0.099
**Sex**^2^			0.242
Male	116 (70%)	485 (66%)	
Female	49 (30%)	255 (34%)	
**Injury type**^2^			**< 0.001**
Blunt	147 (89%)	707 (96%)	
Penetrating	18 (11%)	33 (4%)	
**Arrival GCS**^1^	13 (7, 15)	15 (13, 15)	**< 0.001**
**Arrival vitals**^1^			
Temp	36.6 (36.3, 36.8)	36.7 (36.5, 36.8)	**< 0.001**
SBP	150 (133, 177)	146 (130, 177)	0.599
DBP	89 (75, 100)	89 (77, 100)	0.783
HR	86 (73, 100)	89 (77, 102)	0.079
RR	22 (18, 27)	22 (18, 26)	0.377
O2Sat	99 (96, 100)	98 (95, 100)	**0.037**
**Race**^**2**^			0.462
White	97 (59%)	450 (61%)	
Black	48 (29%)	227 (31%)	
Asian	5 (3%)	10 (1%)	
Other/Unknown	15 (9%)	53 (7%)	

Statistical significance and p-values were assessed for ordinal variables using Wilcoxon rank sum tests^1^ and for categorical variables using Chi-squared tests^2^. GCS = Glasgow coma scale; Temp = temperature (degrees Celcius); SBP = systolic blood pressure (mmHg); DBP = diastolic blood pressure (mmHg); HR = heart rate (beats per minute); RR = respiratory rate (breaths per minute); O2Sat = oxygen saturation (%).

Significant values are in bold.

Inpatient clinical characteristics and hospital discharge outcomes of patients grouped by primary outcome are described in Table 2. Patients who developed ND had a higher incidence of invasive ICP monitoring, higher in-hospital mortality, lower discharge rates to home, and longer hospital lengths of stay (*p* < 0.001). Initial head CT findings are reported in Supplemental Table 2.

**Table 2:** Clinical characteristics and outcomes of hospitalization.

	Neurological decline (N = 905)		*p*-value
	Yes ND, N = 165 (18%)	No ND, N = 740 (82%)	
**Admission destination**^**2**^			
ICU	67 (41%)	112 (15%)	**< 0.001**
IMC	42 (25%)	271 (37%)	
OR	54 (33%)	253 (34%)	
Floor	0 (0%)	68 (9%)	
None/DC	2 (1%)	36 (5%)	
**ICP monitoring**^**2**^	34 (21%)	43 (6%)	**< 0.001**
**Survivor discharge disposition**^**2**^			
Home	23 (18%)	286 (40%)	**< 0.001**
Facility	107 (82%)	427 (60%)	
**Mortality**^**2**^	35 (21%)	27 (4%)	**< 0.001**
**Hospital LOS (days)**^**1**^	9.1 (5.5, 17.0)	5.2 (3.3, 9.0)	**< 0.001**

Statistical significance and p-values were assessed for ordinal variables using Wilcoxon rank sum tests^1^ and for categorical variables using Chi-squared tests^2^. ICP = intracranial pressure; LOS = length of stay; ICU = intensive care unit; IMC = intermediate care unit; OR = operating room; DC = discharged.

Significant values are in bold.

### Model performance and description

**Figure 3:**
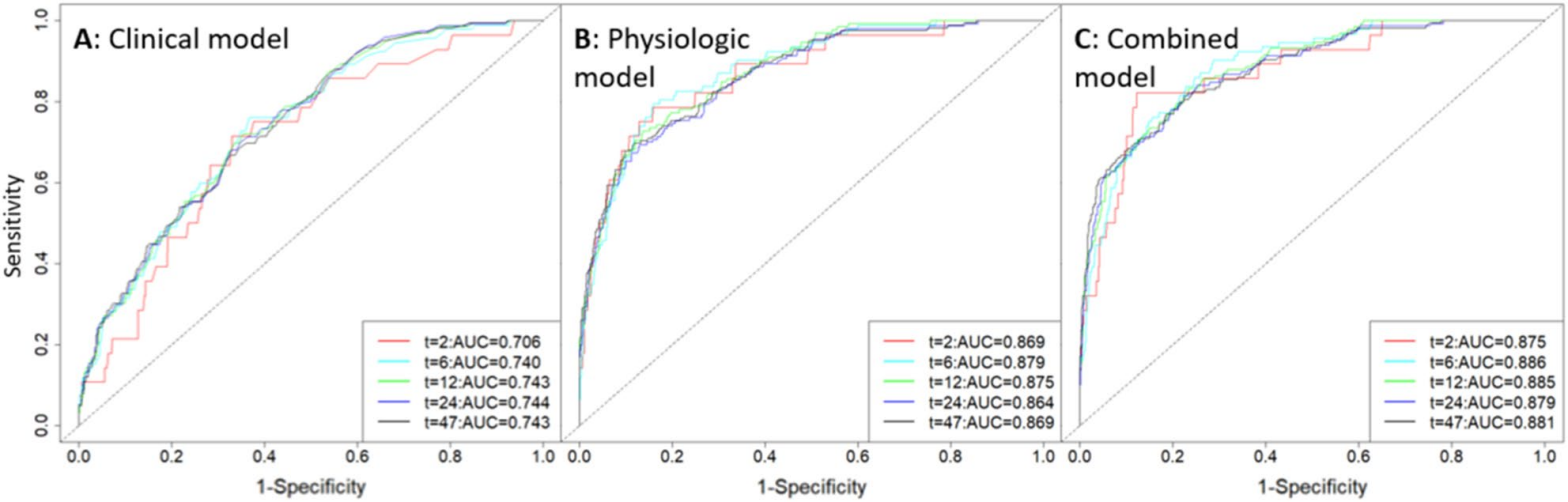
Neurologic decline (ND) prediction model performance based on clinical (**A**), physiologic (**B**), and combined (**C**) predictor variables. Individual receiver operating characteristic (ROC) curves demonstrate model performance for predicting ND at specific times (in hours) from presentation, denoted by line color.

**Figure 4:**
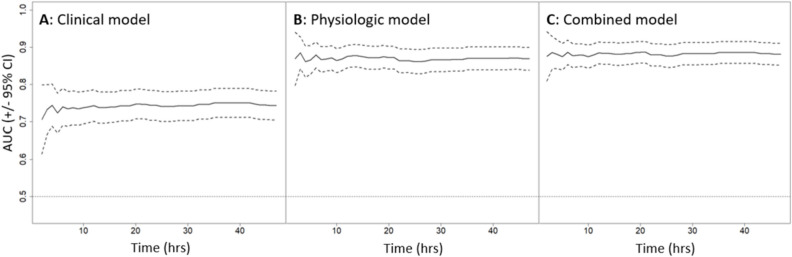
Time-dependent areas under the curve (AUC) of receiver operating characteristic (ROC) analysis (solid lines) displayed with 95% confidence intervals(CI, dotted lines) for ND prediction models based on clinical (**A**), physiologic (**B**), and combined (**C**) predictor variables. The time-independent concordance index (C-Index) for each model was 0.72 (95%CI: 0.69–0.76), 0.84 (95%CI: 0.81–0.87), and 0.85 (95%CI: 0.82–0.88), respectively.

**Figure 5:**
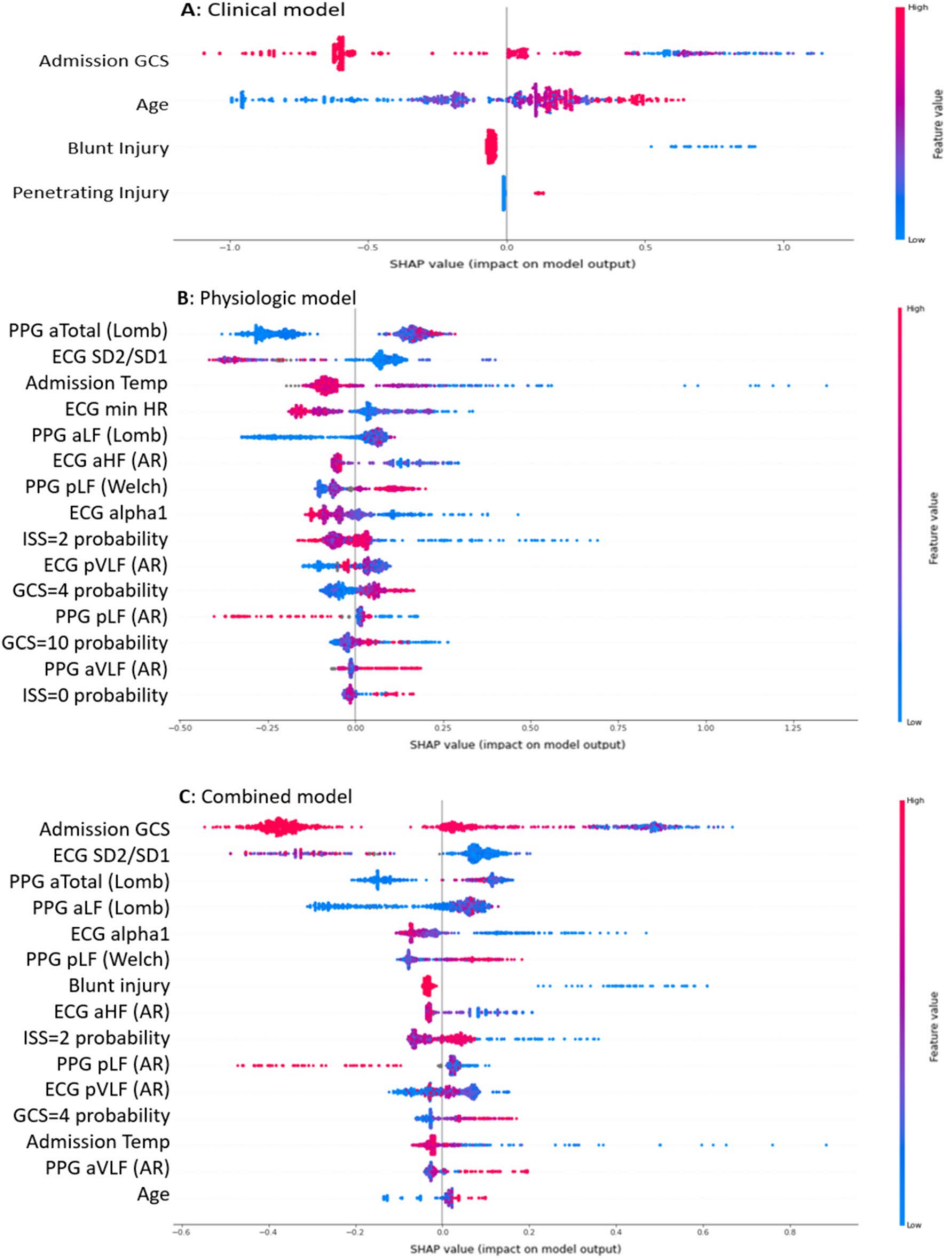
For the clinical (**A**), physiologic (**B**), and combined (**C**) neurologic decline (ND) prediction models, each contributing feature is displayed on the y axis. The x axis shows each feature’s Shapley Additive Explanations (SHAP) values. A larger SHAP value denotes a higher log odds ratio that a variable’s value added to the prediction. Values are represented in color ranging from red to blue (high to low). The y axis from top to bottom ranks the variables’ importance, which is the mean of their absolute SHAP values.

CPH model performance for prediction of ND based on clinical only, physiologic only, and combined features is graphically displayed in Fig. 3. Time-dependent AUCs and 95% confidence intervals are displayed graphically in Fig. 4. The C-index of the physiological model was 0.84 (95%CI: 0.81–0.87), while the C-index of the clinical model was 0.72 (95%CI: 0.69–0.76). The combined model performed similarly to the physiological model, with C-index of 0.85 (95%CI: 0.82–0.88). Our supplemental mortality analysis demonstrated an AUROC of 0.79 (95%CI: 0.73–0.85) in the prediction of in-hospital mortality based on combined ND model output (Supplemental Fig. 1). Contributing feature SHAP values for models are displayed in Fig. 5. In this figure, features are ordered by importance along the y axis, with the most important contributors to the model at the top. Each colored dot represents a single patient’s feature value, with red hue representing higher values and blue hue representing lower values, arranged along the x-axis, according to the log odds ratio that the variable contributed to the model prediction.

## Discussion

In this analysis of 905 TBI patients, we demonstrated that a ML algorithm based on data gathered from the first hour of hospital resuscitation was able to discern time to secondary decline within the first 48 h of admission. As expected, ND was a common clinically meaningful event associated with mortality and worse in-hospital outcomes. This analysis provides early proof of concept that utilizing continuous VS data to develop an automated early warning score for acute TBI patients undergoing inpatient observation may be feasible and has the potential to improve timely triage and resource utilization.

Our model meets several criteria for effective early warning scores^13^—data is gathered primarily electronically, results can be reported in a timely manner, and results may trigger clinical actions to affect a proximate, clinically meaningful outcome. Notably, even our clinical ND models rely on very little manual input, and we included only features that would be readily available on initial presentation. While information such as initial radiographic findings and bloodwork may improve predictive power, we intentionally excluded this information from analysis, since our objective was to identify methods that could also be applied in pre-hospital or austere environments. Our data was collected using wearable sensors already in use as part of standard clinical practice. We generated three rapid ND prediction models in order to improve versatility of application in cases where either physiologic or clinical data is unavailable.

The clinical features contributing to our combined ND prediction model included initial GCS and injury type. GCS is an important contributing variable in existing validated TBI prognostic models including CRASH and IMPACT scores^17,18^. Our finding that lower initial GCS is associated with a higher likelihood of early ND suggests that the relationship between initial GCS and outcome may be not only due to more severe primary injuries, but also to a higher likelihood of secondary ND. While associated with higher mortality, penetrating injury type is less commonly incorporated into TBI prognostic models^46,47^. There is a paucity of literature on the epidemiology and management of penetrating TBI, with some prognostic models excluding this injury type entirely, due to the assumption that it has a distinct pathophysiology. The fact that injury type contributes to our ND prediction model may indicate a need for more focused neuromonitoring in this population. Future work investigating physiologic differences between penetrating and non-penetrating TBI and the efficacy of earlier medical and surgical intervention in salvageable high-risk penetrating TBI patients may be warranted.

Interestingly, older age strongly contributed to increased risk for ND in the clinical model but only weakly contributed in the combined model. Older age consistently has been associated with worse outcomes after TBI^48^, and a recent single center study found a discordance between initial GCS, radiographic severity, and clinical outcome among elderly patients^49^, suggesting that a period of lucidity is more common among older patients who are then more likely to clinically deteriorate. Aging is associated with changes in heart rate variability^50,51^, so while numeric age was a lesser contributor to our combined model, physiologic aging was likely captured by other features.

Physiologic features that contributed to ND prediction included static admission VS, standard HRV features, and some more novel composite physiologic features. The strongest of these included the SD1 to SD2 ratio, the absolute power, and the low frequency power. The SD1 to SD2 ratio is a nonlinear dynamics feature derived from the Poincare plot—a scatterplot comparing each R-R interval to its previous interval^34,52^. It is thought to represent the unpredictability of the beat-to-beat time series and correlates with other HRV measures reflecting the balance between sympathetic and parasympathetic branches of the autonomic nervous system^34^. Our results suggest that a more predictable ECG time series with less physiologic variability is associated with higher likelihood of ND. The total power is a frequency domain feature which represents the variance (average of squared differences from the mean) of all R-R intervals^29^, with our results suggesting that high variance contributed to higher risk for ND in some patients. Low frequency power is another frequency-domain feature that is an accepted marker of sympathetic activity^29,52^, and our results suggest that increased early sympathetic activity was associated with higher likelihood of ND.

Describing all physiologic features contributing to this model is beyond the scope of this work and may become misleading, as the physiologic underpinnings of even the most standard measures remain somewhat opaque. Moreover, our machine learning data-driven model ultimately combines existing parameters into a new clinical-physiologic computational signal associated with ND.

Our physiologic variables included several calculated features derived from transfer learning to estimate the likelihood of clinical variables including GCS and ISS so that the decision support tools could run in a real time automated manner without losing important clinical information. The estimated GCS variables were developed from a large trauma cohort of approximately 28,000 patients^35,36^. This is the first time it has been applied to a TBI-specific population. As the feature’s name implies, it was initially developed as a substitution for GCS in physiologic only models. However, the fact some of the estimated “physiologic GCS” features continue to contribute to the combined model where actual clinical GCS is also included highlights that these features likely capture something unique, beyond GCS. The GCS is a widely used but crude scoring system with a number of limitations including heterogeneity among patients with the same total score. For example, with each GCS total score from 4 to 14, more than one disorder of consciousness is not only possible but occurs with some frequency in the real world, as reported from TRACK-TBI data^53^. One explanation, then, is that the estimated GCS variables may relate more closely to the most frequent level of consciousness associated with each GCS score. Additionally, GCS inter-rater reliability even among neurologists is far from perfect^54^, so we may not expect a perfect correlation between clinical and physiologic GCS. Ongoing work will further evaluate the clinical relevance of these transfer learning features.

Univariable comparisons between patients with versus without ND demonstrated small but statistically significant differences in initial oxygen saturation and body temperature. These values cannot be used in isolation to predict ND. Through multivariable mathematical modeling we found that initial body temperature but not oxygen saturation contributed to ND prediction, as demonstrated in Fig. 5. This is consistent with a pre-hospital study demonstrating that initial body temperature in a large TBI cohort made a difference in predicting mortality^55^. Our findings support the need for machine learning tools to detect subtle but clinically relevant physiologic patterns that would be impossible for a human bedside clinician to recognize.

This is an exploratory proof of concept study with a number of limitations. As the initial phase of a multi-phase study, we aimed to generate robust models predicting neuro-specific decline in the setting of isolated but heterogeneous TBI. To that end, we excluded patients with major thoracic or abdominal trauma that might create systemic sources of ND. It is likely that ND prediction power may improve by stratifying patients based on injury severity, but here we sought a robust signal from TBI patients across the severity spectrum. We hope that our models may eventually serve as a rule-out decision support tool. In this study, we excluded patients who were discharged before 48 h, who were deemed not to require ongoing inpatient monitoring by the clinical team. This resulted in loss of data from the mildest TBI cases, whom we believe unlikely to experience out-of-hospital ND based on conservative observation strategies. Other limitations of this study included retrospective data collection predisposing to reporting bias, which we attempted to minimize by selecting objective and well-documented criteria for ND. While this study included a large cohort that expands upon our previous pilot study work including a mostly mild cohort^19^, prospective and multicenter validation is needed. Without a testing validation cohort, our model may be overfit and performance over-estimated. Our models also do not account for differences in pre-hospital transport times, which were not reliably available for analysis. To address many of these shortcomings, we have begun a more inclusive prospective clinical validation trial (NCT: NCT05084352). Future efforts also may explore the use of ND physiologic prediction models in the pre-hospital setting^16,35^.

We reported model performance here as time-dependent areas under the receiver operating curve, but alternative clinically relevant performance metrics will be explored in the future. As an initial triage decision support tool, we prioritize sensitivity. However, specificity may be improved with the addition of portable and wearable neurosensors, such as reduced montage electroencephalography or pupillometry. However, widespread application of the physiologic models proposed here also will be limited by data infrastructure availability for recording and computing physiologic signals from bedside ECG and PPG monitors.

Further, these models do not represent prescriptive tools; they are capable of providing a risk score for ND but do not suggest a management strategy. This is in contrast to previous work by our group predicting the need for massive transfusion based on early physiologic data^15,35^. At this time, the ND prediction results simply provide early warning notification for near future care planning. Following model validation, simulation-based testing represents a next step; clinicians who work in the trauma resuscitation unit will be given a scenario, the bedside VS display, and a time-dependent ND risk score in order to determine whether and how these scores may affect management practices.

## Conclusions

Data-driven ML models based primarily on non-invasive physiologic monitoring during the “golden hour” of TBI resuscitation have potential to predict risk for subsequent ND and provide an opportunity to mitigate associated secondary injury and worse outcomes. These models may support time-sensitive decisions regarding triage, management, and resource utilization for acute TBI patients, with the goal of improving outcomes and reducing unnecessary costs. The model presented here is based on a large TBI cohort that expands upon previous smaller studies and, if validated, may emerge as an important clinical decision support tool.

## Supplementary Information

Supplementary Information.

## Data Availability

This analysis is part of an ongoing USAF funded study and will be released to FITBIR after the prospective data collection is completed, which is anticipated by July 2024.
