# Supplementary material for: Rapid prediction of secondary neurologic decline after traumatic brain injury: a data analytic approach
Source: Sci Rep. 2023 Jan 9;13:403. doi: 10.1038/s41598-022-26318-4 (PMC9829683; doi:10.1038/s41598-022-26318-4)

Supplemental Information

Rapid prediction of secondary neurologic decline after traumatic brain injury: a data analytic approach

Jamie Podell, Shiming Yang, Serenity Miller, Ryan Felix, Hemantkumar Tripathi, Gunjan Parikh, Catriona Miller, Hegang Chen, Yi-Mei Kuo, Chien Yu Lin, Peter Hu, Neeraj Badjatia

Supplemental Table 1: Heart rate variability features and their definitions

HRV Variable	Definition	Unit
**Time domain**		
**Mean NN**	Mean NN intervals	ms
**SDNN**	Standard deviation of NN intervals	ms
**RMSSD**	Root mean square of the sum of the squares of differences between adjacent NN intervals	ms
**SDNN index**	mean of the SDNN values of each 5-minute segment	ms
**pNN50**	Percentage of NN intervals that differences between adjacent NN intervals > 50ms	%
**pNN20**	Percentage of NN intervals that differences between adjacent NN intervals > 20ms	%
**Frequency domain**		
**Total power**	Spectral power of NN intervals 0-0.4Hz	ms^2
**UVLF**	Spectral power of NN intervals 0 – 0.003Hz	ms^2
**VLF**	Spectral power of NN intervals 0.003-0.04Hz	ms^2
**LF**	Spectral power of NN intervals 0.04-0.15Hz	ms^2
**HF**	Spectral power of NN intervals 0.15-0.4Hz	ms^2
**LF/HF**	Ratio of LF to HF power	
**Nonlinear dynamics**		
**SD1**	Standard deviation of the Poincare plot (PP) perpendicular to the line of identity	ms
**SD2**	SD of the PP along the line of identity	ms
**SD2/SD1**	Ratio of SD2 to SD1	
**Alpha1**	Short term fluctuation slope in detrended fluctuation analysis	
**Alpha2**	Long term fluctuation slope in detrended fluctuation analysis	
**Ellipse area**	Area of the ellipse fit of PP	

Heart rate variability (HRV) features were calculated both from electrocardiography (ECG) and photoplethysmography (PPG) waveforms and were calculated using three different methods, including Welch, Lomb, and autoregressive (AR).

Supplemental Table 2: Initial head computed tomography (CT) features of all included patients, n=905

**Time to CT** (hours)		0.85 [0.52, 1.25]
**Abnormal CT**		388 (42.8%)
**Rotterdam Score**		2 [2, 3]
Basal Cisterns	0 [normal]	720 (90.4%)
	1 [compressed]	46 (5.8%)
	2 [absent]	30 (3.8%)
Midline Shift	0 [< 5mm]	737 (92.6%)
	1 [≥5mm]	59 (7.4%)
Intraventricular or Subarachnoid Hemorrhage	0 [absent]	516 (64.8%)
	1 [present]	280 (35.2%)
Epidural Hematoma	1 [absent]	762 (95.7%)
	0 [present]	34 (4.3%)

Values are reported as either median (1st quartile, 3rd quartile) or as the number (percent of total)

Supplemental Figure 1: Receiver operating characteristic curve exploring neurologic decline model's ability to predict in-hospital mortality

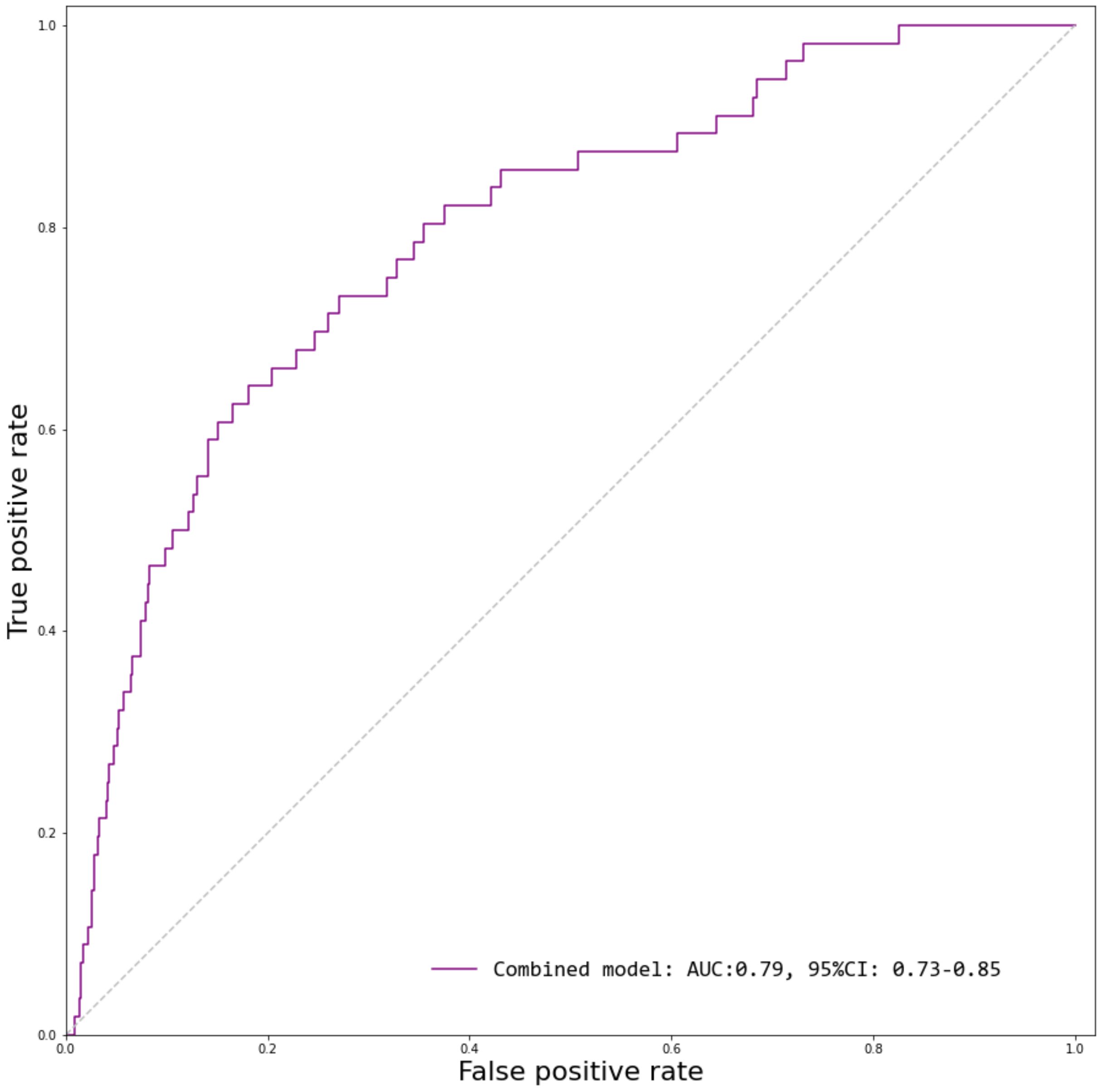

Supplement: Supplementary file 1 — Supplementary Information. [file 41598_2022_26318_MOESM1_ESM.pdf]
